# Fuzzy Entropy-Assisted Deconvolution Method and Its Application for Bearing Fault Diagnosis

**DOI:** 10.3390/e26040304

**Published:** 2024-03-29

**Authors:** Di Pei, Jianhai Yue, Jing Jiao

**Affiliations:** 1 School of Mechanical, Electronic and Control Engineering, Beijing Jiaotong University, Beijing 100044, China; pei_di@bjtu.edu.cn; 2 Locomotive & Car Research Institute, China Academy of Railway Sciences Corporation Limited, Beijing 100081, China; jingjiao@bjtu.edu.cn

**Keywords:** blind deconvolution, fuzzy entropy, fault impulses enhancement, bearing fault diagnosis, vibration analysis

## Abstract

Vibration signal analysis is an important means for bearing fault diagnosis. Affected by the vibration of other machine parts, external noise and the vibration transmission path, the impulses induced by a bearing defect in the measured vibrations are very weak. Blind deconvolution (BD) methods can counteract the effect of the transmission path and enhance the fault impulses. Most BD methods highlight fault features of the filtered signals by impulse-featured objective functions (OFs). However, residual noise in the filtered signals has not been well tackled. To overcome this problem, a fuzzy entropy-assisted deconvolution (FEAD) method is proposed. First, FEAD takes advantage of the high noise sensitivity of fuzzy entropy (FuzzyEn) and constructs a weighted FuzzyEn–kurtosis OF to enhance the fault impulses while suppressing noise interference. Then, the PSO algorithm is used to iteratively solve the optimal inverse deconvolution filter. Finally, envelope spectrum analysis is performed on the filtered signal to realize bearing fault diagnosis. The feasibility of FEAD was first verified by the bearing fault simulation signals at constant and variable speeds. The bearing test signals from Case Western Reserve University (CWRU), the railway wheelset and the test bench validated the good performance of FEAD in fault feature enhancement. A comparison with and quantitative results for the other state-of-the-art BD methods indicated the superiority of the proposed method.

## 1. Introduction

Rolling element bearings are widely used in rotating machinery for major equipment such as electric multiple units (EMU), airplanes and wind turbines. Beatings are prone to failure due to harsh operating environments like high speeds, heavy loads, high temperatures and so on. Once the bearing malfunctions, it will cause damage to mechanical equipment or even lead to catastrophic accidents. Therefore, it is of great significance to intervene in advance by accurately diagnosing bearing faults.

Vibration signal analysis is the most common means of bearing fault diagnosis, as bearing vibration signals contain rich status information and are easy to obtain. The measured bearing fault signal can be considered as the convolution operation performed on fault impulses and the transfer function of the path between the fault source and the sensor [1]. Therefore, the fault impulses are weakened in the vibration signal measured by sensors. Extracting weak fault features from the measured vibrations has become a research focus. In recent years, a large number of advanced and effective methods have been proposed to enhance fault features from bearing vibration signals, such as wavelet transform [2], adaptive signal decomposition methods [3,4,5], spectral kurtosis [6,7], fuzzy logic related methods [8,9] and BD methods [10,11,12]. Among them, the BD methods can counteract the influence of the transmission path by adaptively designing an optimal finite impulse response (FIR) filter [1,13]. Compared with other methods, BD methods determine the optimal filter adaptively based on the characteristics of the signal without being limited by the center frequency and bandwidth, and so they have been successfully applied in rotating machinery fault diagnosis.

In 2007, Endo and Randall [10] first presented minimum entropy deconvolution (MED) to extract fault features of rotating machinery and showed good results. MED uses kurtosis as the deconvolution OF and iteratively updates the filter coefficients to make the filtered signal restore the fault impulses with the maximum kurtosis value. However, MED prefers to deconvolute a large random impulse, which is inconsistent with the multi-impulse characteristic of the bearing fault signal. To overcome the limitation of MED, McDonald et al. [11] proposed maximum correlated kurtosis deconvolution (MCKD) with correlated kurtosis as the OF to enhance the periodic fault impulses from the measured vibration signal at constant speed (CS). However, the effect of MCKD is greatly affected by the input parameters. Only with accurate period and appropriate shift orders can continuous fault impulses be deconvoluted. In addition, both MCKD and MED optimize the filter coefficients in an iterative manner by taking partial derivatives of the OF, which may lead the filter results to fall into a local optimum rather than the global optimal solution. In order to alleviate the shortcomings of MED and MCKD, McDonald and Zhao [12] first proposed MED adjusted (MEDA) to avoid the discontinuity between the two sides of the filtered signal. Then, a non-iterative BD method called multipoint optimal MEDA (MOMEDA) was proposed in their work. MOMEDA introduces a target vector to determine the location and the weight of fault impulses, and solves the optimal filter directly by taking multi D-norm as the deconvolution OF. The non-iterative manner allows MOMEDA to obtain the optimal solution rather than the good one. Meanwhile, MOMEDA can output a more complete train of impulses. Nevertheless, similar to MCKD, as a period-based BD method, MOMEDA’s performance strongly relies on prior knowledge of the fault period, which makes it unable to handle non-stationary bearing signals measured under variable speed (VS) directly. Meanwhile, the effect of MOMEDA was reported susceptible to the length of the filter [13,14].

There are many improved methods proposed to address the issues mentioned above. These improvements can be categorized into five aspects: (**1**) New OFs were constructed to depict the fault information comprehensively. Miao et al. [15] proposed the sparse maximum harmonics–noise–ratio (HNR) deconvolution method, which solves the optimum filter to maximize the HNR of the output signal. Buzzoni et al. [16] proposed to use indicators of cyclostationarity as the OF in the deconvolution problem and called it the maximum second-order cyclostationarity blind deconvolution (CYCBD). Liang et al. [17] proposed a new index named average kurtosis (AK), and the corresponding BD method is called maximum average kurtosis deconvolution (MAKD). Hashim and Shakya [18] treated the angular domain spectral kurtosis as the OF and proposed the spectral kurtosis deconvolution (SpKD) approach for gear fault diagnosis. (**2**) New approaches for solving filter coefficients were proposed to replace the iterative solution methods. Cheng et al. [14,19] used the particle swarm optimization (PSO) algorithm to solve the deconvolution filters and proposed the PSO-MED, PSO-MCKD and PSO-MOMEDA methods. Experimental results showed that they have better performance than the traditional BD methods at a low signal-to-noise ratio (SNR) and can reduce the dependence on the input parameters. Jia et al. [20] employed multi-layer neural networks to solve the optimal filter in the deconvolution problem. (**3**) The input parameters of BD methods were optimized rather than preset empirically. PSO [13], grid search [21] and periodic modulation intensity [22] were used to obtain the length of filter and the period of the target vector in MOMEDA. Miao et al. [23] estimated the period of MCKD by calculating the autocorrelation of the envelope signal. MAKD [17] employed an optimized Morlet wavelet as the initial filter, aiming at improving the computational efficiency. (**4**) Equal-angle resampling was employed as a preprocessing method to adapt BD methods to the vibration signal under VS. For example, MOMEDAang [24], MAKD [17] and SpKD [18] resample the original vibrations to the angular domain and then perform deconvolution. (**5**) BD was combined with other methods to achieve better fault feature enhancement effects, for instance, the combination of MOMEDA with EEMD [21], VMD [25], resonance-based sparse signal decomposition (RSSD) [13] and Ramanujan subspace decomposition [26], and the combination of MED with SK [27].

The bearing fault vibration signals collected from industrial sites, especially under incipient damage and VS conditions, show weak fault characteristics and are prone to be submerged in ambient noise. Although MED, MCKD, MOMEDA and their improvements have been widely used in bearing fault diagnosis, these methods are designed to enhance the periodic impulse feature while ignoring the residual noise in the filtered signal. Meanwhile, the error introduced by equal-angle resampling will degrade the performance of the period-based BD methods when processing the non-stationary bearing vibration signals.

In order to overcome the influence of noise and non-stationary operating conditions, a new BD method called fuzzy entropy-assisted deconvolution is proposed for bearing fault feature enhancement while suppressing the noise in the filtered signal. FEAD is based on MEDA and takes advantage of the high noise sensitivity of FuzzyEn. By adding the FuzzyEn auxiliary term to the kurtosis-dominated OF, a weighted FuzzyEn–kurtosis OF is constructed for the deconvolution problem. The standard PSO algorithm is employed to iteratively solve the global optimal FIR filter, which minimizes the FuzzyEn–kurtosis of the filtered signal. Bearing fault diagnosis can then be realized by the envelope spectrum or order envelope spectrum of the filtered signal. The feasibility of FEAD was first verified through bearing fault simulation signals at CS and VS. Then, the reliability and the effectiveness of FEAD were validated using the bearing ball fault signal from CWRU, the bearing inner race fault signal at CS from railway wheelset, and the bearing outer race fault signal at VS from the EMU transmission test bench, respectively. Simulation and experimental results show that bearing faults can be accurately diagnosed thanks to the fact that FEAD can enhance the fault impulse feature and suppress the noise simultaneously. Comparisons with and quantitative analyses of other popular BD methods, i.e., MEDA, PSO-MEDA, MCKD and MOMEDA, reveal the following advantages of the proposed method: First, FEAD has better performance in suppressing the interference of ambient noise than other BD methods because the noise sensitivity of FuzzyEn is higher than that of kurtosis. Second, FEAD is a non-periodic-based deconvolution method, which means it can directly process bearing vibration signal at VS so that the error from equal-angle resampling can be avoided.

The originality of this paper is that we extend the entropy method to blind deconvolution, so that the objective functions in the BD method can be a combination of multiple items rather than a single one. This can help the BD methods achieve more comprehensive performance, especially in processing bearing vibration signals from complex mechanical system.

The rest of this paper is organized as follows: Section 2 overviews MEDA, FuzzyEn and standard PSO methods. Section 3 first investigates the noise sensitivity of kurtosis and entropy indicators, and then introduces the proposed FEAD and its detailed implementation procedure for bearing fault diagnosis. Section 4 compares and quantitatively evaluates FEAD with MEDA, PSO-MEDA, MCKD and MOMEDA based on bearing simulation signals at CS and VS. In Section 5, the actual bearing signals from CWRU, railway wheelset and the EMU transmission test bench verify the reliability and effectiveness of FEAD. Comparisons and quantitative analyses are also given to validate the superiority of the proposed method. Conclusions are drawn in Section 6.

## 2. Theoretical Background

### 2.1. MEDA

MED is the first applied BD method for the fault diagnosis of rotating machinery [10] and can be used to process bearing vibration signals at VS directly [17]. MED is designed to counteract the transmission path effect and maximize the kurtosis of the filtered signal by iteratively optimizing the FIR filter.

Let ***x*** = [*x*_1_, *x*_2_, …, *x_N_*]^T^ be the measured vibration signal that contains noise and other interference, such as random impulses caused by external knock and harmonics from other rotational parts. The element in the filtered signal can be produced by convolving ***x*** with an FIR filter ***f*** = [*f*_1_, *f*_2_, …, *f_L_*]^T^, written as follows:

(1) $$y_{k}=\sum_{l=1}^{L}f_{l}x_{k+L-l},\;k=1,2,\ldots ,N-L,$$

The adjusted convolution definition in Equation (1) avoids spurious impulses at the endpoints of the filtered signal ***y*** = [*y*_1_, *y*_2_, …, *y_N_*_−*L*_]^T^. The MED defined with Equation (1) is called MEDA [12]. In order to recover the impulses component from the measured signal as much as possible, MEDA takes the kurtosis as the OF to iteratively solve the following inverse filter:

(2) $$\hat{f}=\underset{f}{\arg\max}[O_{MEDA}(y)],$$

where $\hat{f}$ represents the estimation of the optimal filter. *O_MEDA_*(·) is the OF of MEDA:

(3) $$O_{MEDA}(y)=\frac{\sum_{k=1}^{N-L}y_{k}^{4}}{{\left(\sum_{k=1}^{N-L}y_{k}^{2}\right)}^{2}}$$

Taking the derivatives of Equation (3) with respect to filter coefficients ***f*** and solving it equal to zero:

(4) $$\frac{\partial O_{MEDA}(y)}{\partial f}=0,$$

The optimal value of ***f*** can be iteratively solved with the following equation:

(5) $$\hat{f}=\frac{\sum_{k=1}^{N-L}y_{k}^{2}}{\sum_{k=1}^{N-L}y_{k}^{4}}{\left(X_{0}X_{0}^{T}\right)}^{-1}X_{0}{\left[\begin{array}{llll} y_{1}^{3} & y_{2}^{3} & \ldots & y_{N-L}^{3} \end{array}\right]}^{T},$$

where

(6) $$X_{0}={\left[\begin{array}{cccc} x_{L} & x_{L+1} & \cdots & x_{N-1}\\ x_{L-1} & x_{L} & \cdots & x_{N-2}\\ \vdots & \vdots & \ddots & \vdots \\ x_{1} & x_{2} & \cdots & x_{N-L} \end{array}\right]}_{L\;\mathrm{by}\;N-L}$$

The iterative solution starts with an initial filter ***f*** = [0, …, 0, 1, −1, 0, …, 0]^T^, then updates ***f*** through Equation (5) based on the filtered signal ***y*** obtained from Equation (1) until the iteration number reaches the preset value or a minimum change in the kurtosis of the filtered signals between iterations. Finally, the filtered output signal can be calculated by the convolution of ***x*** and $\hat{f}$ in matrix form:

(7) $$y=X_{0}^{T}\hat{f}$$

### 2.2. Fuzzy Entropy

Entropy is a statistical measure, it can quantify the complexity and detect dynamic changes in time series and has been widely applied in bearing fault diagnosis [28,29]. Among various entropy methods, FuzzyEn is a popular entropy calculation method that was utilized in bearing fault detection and classification [30]. The specific calculation steps of FuzzyEn are listed as below [31].

Obtain the coarse-grained sequence $X_{i}^{m}$ from the original signal sequence ***x*** based on Equation (8):

(8) $$\left\{\begin{array}{c} X_{i}^{m}=[x(i),x(i+1),\ldots ,x(i+m-1)]-x_{0}(i),\;i=1,\ldots ,N-m+1\\ x_{0}(i)=\frac{1}{m}\sum_{l=0}^{m-1}x(i+l) \end{array}\right.,$$

where *N* is the length of ***x***; *m* is the length of coarse-grained sequence. The value of *m* is usually taken as 2.

Calculate the maximum absolute distance $d_{ij}^{m}$ between $X_{i}^{m}$ and $X_{j}^{m}$:

(9) $$d_{ij}^{m}=d[X_{i}^{m},X_{j}^{m}]=\underset{k\in (0,m-1)}{\max}\left|\left(x(i+k)-x_{0}(i)\right)-\left(x(j+k)-x_{0}(j)\right)\right|,\;j\neq i$$

Given *n* and *r*, calculate the similarity degree $D_{ij}^{m}\;$of $X_{i}^{m}$ to $X_{j}^{m}$ through an exponential fuzzy function:

(10) $$D_{ij}^{m}(n,r)=u(d_{ij}^{m},n,r)=\exp\left(-{\left(d_{ij}^{m}\right)}^{n}/r\right),$$

The parameters *r* and *n* in Equation (10) determine the width and the gradient of boundary for the fuzzy function, and their values are set as 0.2 and 2, respectively.

Define the function *ϕ^m^* as follows:

(11) $$\phi^{m}(n,r)=\frac{1}{N-m}\sum_{i=1}^{N-m}\left(\frac{1}{N-m-1}\sum_{j=1,j\neq i}^{N-m}D_{ij}^{m}\right)$$

Similarly, form $X_{i}^{m+1}$ and obtain the function *ϕ^m^*^+1^

(12) $$\phi^{m+1}(n,r)=\frac{1}{N-m}\sum_{i=1}^{N-m}\left(\frac{1}{N-m-1}\sum_{j=1,j\neq i}^{N-m}D_{ij}^{m+1}\right)$$

Finally, for finite datasets, the FuzzyEn of ***x*** can be calculated based on *ϕ^m^* and *ϕ^m^*^+1^:

(13) $$FuzzyEn(x)=\ln\phi^{m}(n,r)-\ln\phi^{m+1}(n,r)$$

### 2.3. PSO Algorithm

As a population-based global optimization algorithm, PSO is inspired by the social behaviour of bird flocks looking for corn [32]. The standard PSO 2011 (SPSO-2011) [32,33] showed outstanding performance over previous PSO versions and has been used for solving the filter coefficient in BD methods [14,19]. Therefore, SPSO-2011 is used to solve the optimization problem in the proposed FEAD.

In the PSO algorithm, each individual in the population is known as a particle. The particle adjusts the flying trajectory in the search space according to its own previous flying experience and the neighbouring particles in the swarm.

In a search space that defined as the Euclidean product of *D* real intervals

(14) $$E=\overset{D}{\underset{d=1}{⊗}}[min_{d},max_{d}],$$

vectors ***X****_i_* = [*x_i_*_1_, *x_i_*_2_, …, *x_iD_*]^T^ and ***V****_i_* = [*v_i_*_1_, *v_i_*_2_, …, *v_iD_*]^T^ are used to represent the position and velocity of the *i*th particle, respectively. A problem-specific fitness function to be minimized is defined as *F*(·); it is the performance assessment and the basis for updating each particle. The optimization problem is summarized as follows:

(15) $$\begin{array}{l} \mathrm{Given}\;F(\cdot ):\;E\to ℜ\\ \mathrm{Find}\;X_{opt}|F(X_{opt})\leq F(X_{i})\;\forall X_{i}\in E \end{array}$$

The optimal solution to the problem relies on the movement of particles to the optimal position in the search space. The *i*th particle’s position is updated using the following equation:

(16) $$X_{i}^{t+1}=X_{i}^{t}+V_{i}^{t+1},$$

where *i* = 1, 2, …, *S*, with *S* equal to the swarm size; and *t* = 1, 2, …, *T*, with *T* equal to the total number of iterations. In present paper, *S* and *T* are set as floor(10 + 2$\sqrt{D}$) and 100, respectively. The particle’s new position $X_{i}^{t+1}$ can be updated by a linear combination of its old position and new velocity. The particle’s new velocity is updated according to the following equation:

(17) $$V_{i}^{t+1}=\omega V_{i}^{t}+x_{i}^{\prime }-X_{i}^{t},$$

where *ω* is the inertia weight that aims to prevent swarm explosion, and 1/(2ln(2)) is recommended. $x_{i}^{\prime }$ is a random point defined in the hypersphere $H_{i}(G_{i}^{t},\;\left|\right|G_{i}^{t}-X_{i}^{t}\left|\right|)$ of centre $G_{i}^{t}$ and of radius $\left|\right|G_{i}^{t}-X_{i}^{t}\left|\right|$. During the iteration, the personal best that is represented as ***P****_i_* = [*p_i_*_1_, *p_i_*_2_, …, *p_iD_*]^T^ is the best-known position of the *i*th particle, whereas the local best that is represented as ***L*** = [*l*_1_, *l*_2_, …, *l_D_*]^T^ means the best-known position within the particle’s neighborhood. The detailed definition of a particle’s neighborhood and swarm topology that controls the exchange of information between particles can be found in [34]. Assuming that the neighbors consists of *K* particles, the local best is defined as follows:

(18) $$L^{t}=\underset{j=1,\;2,\;\ldots ,\;K}{\arg\min}F(P_{j}^{t})$$

For each particle at the *t*-th iteration, three points, i.e., the current position, a point a little “beyond” the previous personal best, and a point a little “beyond” the previous local best, are employed to define a centre of gravity $G_{i}^{t}$:

(19) $$G_{i}^{t}=\frac{X_{i}^{t}+(X_{i}^{t}+c_{1}(P_{i}^{t}-X_{i}^{t}))+(X_{i}^{t}+c_{2}(L^{t}-X_{i}^{t}))}{3},$$

*c*_1_ and *c*_2_ are the cognitive and social acceleration coefficients, and their values are recommended as *c*_1_ = *c*_2_ = 1/2 + ln2 in reference [33].

The global best particle ${Gl}^{t}$ and the personal best particle are updated in each iteration as follows:

(20) $$Gl^{t}=\underset{i=1,\;2,\;\ldots ,\;S}{\arg\min}F(P_{i}^{t})$$

(21) $$P_{i}^{t+1}=\left\{\begin{array}{l} X_{i}^{t+1},\;F(X_{i}^{t+1})\leq F(P_{i}^{t})\\ P_{i}^{t},\;F(X_{i}^{t+1})>F(P_{i}^{t})\; \end{array}\right.$$

The global optimal particle ${Gl}^{T}$ obtained in the last iteration is the solution to the optimization problem.

In addition, in SPSO-2011, ***X****_i_*, ***V****_i_* and ***P****_i_* are initialized as follows:

(22a) $$X_{i}^{0}=U(min_{d},max_{d})$$

(22b) $$V_{i}^{0}=U(min_{d}-x_{id}^{0},max_{d}-x_{id}^{0})$$

(22c) $$P_{i}^{0}=X_{i}^{0}$$

*U*(·) in Equation (22a–c) represents the uniformly distributed random numbers.

## 3. Proposed Fuzzy Entropy-Assisted Deconvolution Method

FEAD is proposed based on the fact that fuzzy entropy is more sensitive to noise than kurtosis. This advantage is beneficial to alleviating residual noise interference in the deconvolution output signal. Starting from the bearing vibration signal model, this section investigates the sensitivity of different feature indicators to the noise in bearing signals, and then proposes the FEAD method for bearing fault diagnosis.

### 3.1. Bearing Vibration Signal Model

When a single local damage occurs in bearing, there are four frequently encountered signal components in the measured vibration [14,23]:

(23) x(t)=b(t)+d(t)+h(t)+n(t)

The first part represents the impulse component caused by the bearing fault. When a bearing works at CS, the periodic impulses can be modeled as follows:

(24) $$b_{c}(t)=\sum_{i=1}^{N}A_{ic}e^{-\beta_{1}\left(t-iT-\tau_{i}\right)}\sin\left[2\pi \omega_{r1}\left(t-iT-\tau_{i}\right)\right]u\left(t-iT-\tau_{i}\right),$$

where *N* and *A_ic_* are the total number of impulses and the amplitude of the *i*th impulse at CS; *T* specifies the nominal time interval between two adjacent fault impulses, and the relationship between *T* and fault characteristic frequency (FCF) is *T* = 1/*FCF*; *τ_i_* ∈ [0.01, 0.02]*T_t_*/*N* represents the time error of the impulses caused by the bearing slippage, where *T_t_* is the total duration of the signal; *ω_r_*_1_ and *β*_1_ denote the resonant frequency and the decay parameter excited by fault, respectively; and *u*(‧) represents the unit step function.

When a bearing works at VS, the time intervals between fault impulses are not constant. Therefore, modified from Equation (24), the non-periodic impulses can be modeled as follows:

(25) $$b_{v}(t)=\sum_{i=1}^{N}A_{iv}e^{-\beta_{1}\left(t-T_{i}-\tau_{i}\right)}\sin\left[2\pi \omega_{r1}\left(t-T_{i}-\tau_{i}\right)\right]u\left(t-T_{i}-\tau_{i}\right),$$

where *A_iv_* is the amplitude of the *i*th impulse at VS. This paper assumes that the amplitude of the fault impulses change linearly with the rotational frequency (RF) [35], that is, *A_iv_* = *A*_0_ + *ηf_r_*(*T_i_*). *A*_0_ is a constant, *η* is a proportional coefficient, and *f_r_*(*T_i_*) represents the RF of the bearing at the *i*th fault impulse. The relationship between *T_i_* and the RF *f_r_*(*t*) (in Hz) can be expressed as follows:

(26) $$\int_{0}^{T_{i}}f_{r}(t)dt=\frac{i}{FCC},i=1,2,\cdots ,N$$

*FCC* is the fault characteristic coefficient. Hence, *T_i_* can be solved by the numerical method from Equation (26).

The second part represents the random impulse component derived from external knocks on the housing, which can be formulated as follows:

(27) $$d(t)=\sum_{m=1}^{M}D_{m}e^{-\beta_{2}\left(t-T_{m}\right)}\sin\left[2\pi \omega_{r2}\left(t-T_{m}\right)\right]u\left(t-T_{m}\right),$$

where *M* represents the number of random impulses; *D_m_* and *T_m_* are the amplitude and the occurrence time of the *m*th random impulse, respectively. *ω_r_*_2_ and *β*_2_ denote the resonant frequency and the decay parameter excited by random impulses, respectively.

The second part represents the discrete harmonic component caused by other rotating parts such as shaft and gears, which can be modeled as follows:

(28) $$h(t)=P_{1}\sin(2\pi \int h_{1}(t)dt+\theta_{1})+P_{2}\sin(2\pi \int h_{2}(t)dt+\theta_{2}),$$

where *P*_1_ (*P*_2_), *h*_1_ (*h*_2_) and *θ*_1_ (*θ*_2_) represent the amplitude, frequency and initial phase of the shaft rotating (gear meshing) harmonic, respectively.

The fourth part *n*(*t*) in Equation (23) represents the ambient noise with a normal distribution.

It is worth noting that the vibration components in actual mechanical system are much more complex than those in the model. The above model inevitably has some limitations. For example, the amplitude modulation caused by inner race or rolling element faults, and the amplitude differences due to fault type and size are not considered.

### 3.2. Proposal of FEAD

The OF plays an important role in the BD method. An OF that can simultaneously highlight fault impulses and suppress noise interference is beneficial to recovering the fault impulse characteristic from the measured vibration signals. Although named minimum entropy deconvolution, MED does not use entropy but kurtosis as the OF. A high kurtosis value is only one of the necessary conditions for fault impulses rather than a sufficient condition, and this is the root cause why MED fails under certain conditions. Entropy is another important indicator that measures the intensity of the fault in the vibration signal. Generally, the stronger the fault impulses in the bearing vibration signal, the higher the kurtosis value and the lower the entropy value, and vice versa. However, entropy and kurtosis do not simply vary in inverse proportions. They have dissimilar sensitivities to different components in the bearing vibration signal.

In order to elucidate the motivation of FEAD, based on the above-mentioned bearing vibration models at CS and VS, the sensitivities of entropy and kurtosis to different signal components are investigated. The specific model parameters are listed in Table 1, where *h_c_*(*t*) and *h_v_*(*t*) represent the harmonic components at constant and variable speeds, respectively. The sampling frequency of simulation signals is 12,000 Hz.

**Figure 1:**
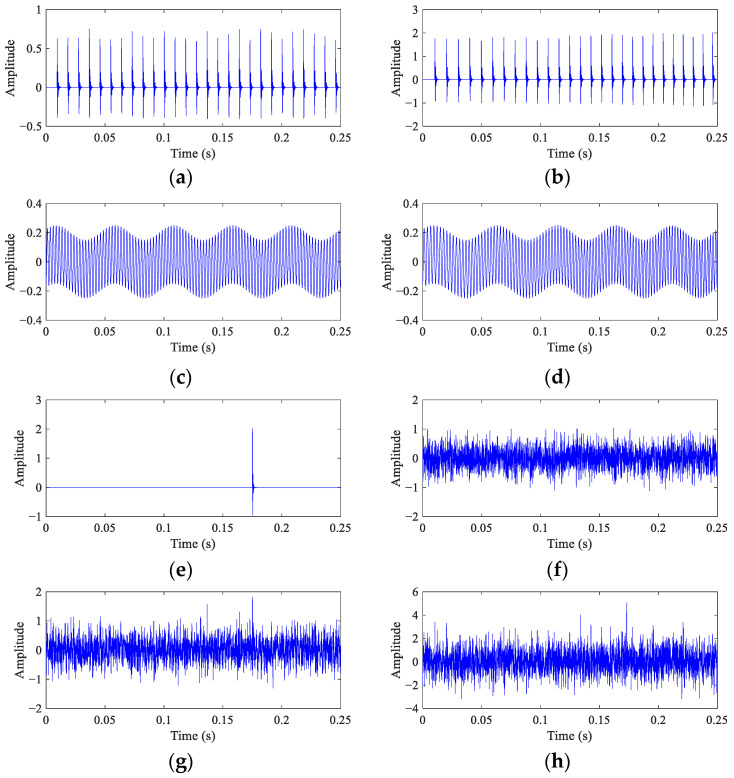
The components and the mixed signals from bearing vibration models. (**a**,**b**) Fault impulses at CS and VS; (**c**,**d**) discrete harmonics at CS and VS; (**e**) random impulse; (**f**) white noise with *std* = 0.35; (**g**,**h**) mixed signals at CS and VS.

The waveforms of different components and mixed signals are shown in Figure 1. It can be seen that the interval of impulses and the frequency of harmonic remain unchanged at CS (Figure 1a,c), while they change with the RF at VS (Figure 1b,d). The amplitude of random impulse displayed in Figure 1e is higher than the fault impulses in Figure 1a,c. The fault impulses are submerged in the noise, while the random impulses are significant in the mixed signals (Figure 1g,h).

For a more comprehensive analysis, in addition to FuzzyEn and kurtosis, approximate entropy (ApEn) and sample entropy (SampEn) are also used for comparison. The formula for calculating the kurtosis of a discrete signal *y_n_* (*n* = 1, 2, …, *N*) is as follows:

(29) $$Kurt(y_{n})=\frac{\frac{1}{N}\sum_{n=1}^{N}{\left(y_{n}-\bar{y}\right)}^{4}}{{\left(\frac{1}{N}\sum_{n=1}^{N}{\left(y_{n}-\bar{y}\right)}^{2}\right)}^{2}},$$

where $\bar{y}$ is the mean value of *y_n_*. The calculation methods for ApEn and SampEn can refer to [36].

Table 2 lists the kurtosis and entropy values of different components and mixed signals at CS. For the kurtosis indicator, the random impulse has the largest value, followed by continuous fault impulses, and the smallest is the harmonic component. MED aims to maximize the kurtosis of the output signal, and so it is prone to deconvolute a single random impulse than continuous fault impulses, while harmonic interference can be effectively suppressed. For the FuzzyEn indicator, the random impulse has the smallest value, followed by harmonics and fault impulses. Therefore, if the deconvolution filter is optimized by minimizing the FuzzyEn of the output signal, it is easy to deconvolute the random impulse and the harmonic components. On the contrary, the fault impulses may be suppressed to a certain extent. The sensitivities of ApEn and SampEn to *b_c_*(*t*), *d*(*t*) and *h_c_*(*t*) are similar to kurtosis (assuming maximizing kurtosis and minimizing entropy).

Table 3 lists the kurtosis and entropy values of different components and mixed signals at VS. It can be concluded that the sensitivities of kurtosis and entropy indicators to *b_v_*(*t*), *d*(*t*) and *h_v_*(*t*) are similar to those at CS.

The above analysis discovers the sensitivity of kurtosis and entropy indicators to fault impulses, random impulses and harmonic components. More importantly, the BD method should effectively suppress the noise interference in the output signal. In order to quantify the sensitivity of kurtosis and entropy indicators to noise, Gaussian white noise with different variances are added to the noise-free signal, denoted as *x_free_* = *b*(*t*) + *d*(*t*) + *h*(*t*). The relative change rates of different indicators from the noisy signal, denoted as *x_noisy_* = *x_free_* + *n*(*t*), to the noise-free signal are employed as a noise sensitivity measure:

(30) $$\Delta I=\frac{\left|Idc(x_{noisy})-Idc(x_{free})\right|}{Idc(x_{noisy})}\times 100\%$$

**Figure 2:**
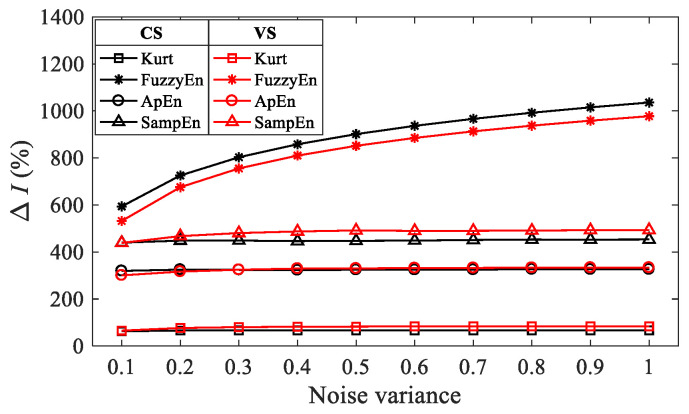
The change rate of kurtosis and entropy indicators at different noise levels.

In Equation (30), *Idc*(·) represents the calculation of different indicators, namely *Kurt*(·), *FuzzyEn*(·), *ApEn*(·) and *SampEn*(·). The higher the change rate of a certain indicator, the stronger its sensitivity to noise, which means a better noise suppression effect when treating it as the OF in BD method. Herein, the variance of noise is increased from 0.1 to 1 in steps of 0.1 to simulate different noise levels. Figure 2 illustrates the change rate of kurtosis and entropy indicators for CS and VS models at different noise levels.

It can be concluded from Figure 2 that the change rate of entropy indicators is greater than the kurtosis for both speed conditions. FuzzyEn is more sensitive to noise than ApEn and SampEn. As the noise variance increases, the noise sensitivity of kurtosis, ApEn and SampEn remain almost unchanged, while the noise sensitivity of FuzzyEn shows an increasing trend.

The FEAD method is inspired by the above-mentioned sensitivity of kurtosis and entropy indicators to different components and noise in the bearing vibration signal. The purpose of FEAD is to enhance the fault impulse component while further suppressing the noise interference in the deconvolution signal. Therefore, taking the advantage of the high sensitivity of kurtosis to fault impulses and FuzzyEn to noise, FEAD constructs a weighted FuzzyEn-Kurt indicator as the deconvolution OF:

(31) $$O_{FEAD}(y)=\alpha FuzzyEn(y)-\beta \mathrm{lg}[Kurt(y)],$$

where lg[·] represents the common logarithm with base 10, it acts on kurtosis term to narrow the magnitude gap between kurtosis and FuzzyEn. *α* and β with a range [0, 1] weight the FuzzyEn term and the Kurt term in the OF, respectively.

To ensure that the filtered signal satisfies high kurtosis and low FuzzyEn simultaneously, FEAD solves the deconvolution filter by minimizing the new objective function. The corresponding deconvolution problem can be expressed as follows:

(32) $$\hat{f}=\underset{f}{\arg\min}[O_{FEAD}(y)]$$

When *α* = 0, FADE degrades to MEDA; when β = 0, FADE degrades to “minimum fuzzy entropy deconvolution”.

### 3.3. Procedure of FEAD for Bearing Fault Diagnosis

**Figure 3:**
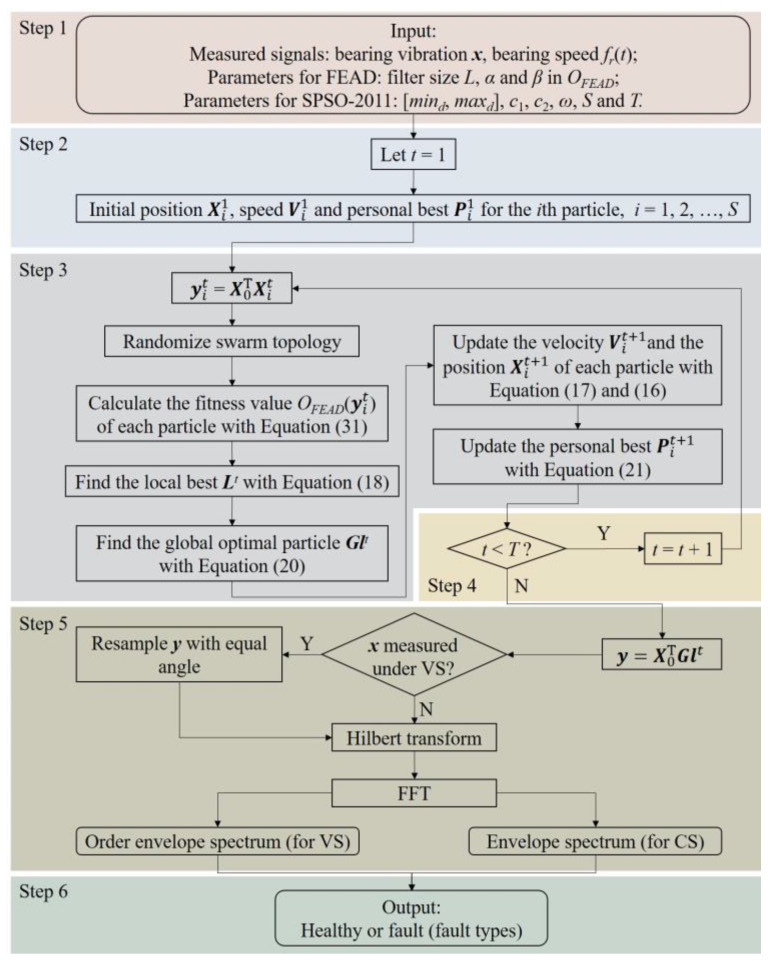
Flowchart of the proposed FEAD bearing fault diagnosis method.

Since the OF in FEAD is complex and nonlinear, it is hard to solve the deconvolution problem like MEDA. Therefore, FEAD employs PSO to optimize the filter coefficients. The flowchart of the bearing fault diagnosis method based on FEAD is illustrated in Figure 3.

The specific steps are listed as follows:

1. Input the measured bearing signals, the parameters of SPSO-2011 and FEAD.
2. Let *t* = 1; for each particle, initialize its position $X_{i}^{1}$, speed $V_{i}^{1}$ and personal best $P_{i}^{1}$ (*i* = 1, 2, …, *S*) based on Equation (22).
3. Perform the convolution operation between ***x*** and $X_{i}^{1}$ to obtain the filtered signal $y_{i}^{t}$. Randomize the swarm topology and calculate the fitness value *O_FEAD_*($y_{i}^{t}$) of each particle using Equation (31). Based on the fitness value of the particle and its neighbors determined by the topology, find the local best ***L****^t^* and the global optimal particle ***Gl****^t^* using Equations (18) and (20), respectively. Then, update the velocity $V_{i}^{t+1}$ and the position $X_{i}^{t+1}$ of each particle using Equations (17) and (16), respectively. Finally, update the personal best $P_{i}^{t+1}$ for the next iteration.
4. If the number of iterations is less than the preset value, let *t* = *t* + 1 and repeat step 3. Otherwise, proceed to the next step.
5. Treat the global optimal ***Gl****^T^* as the optimal filter coefficient, convolve it with ***x*** to obtain impulses’ enhanced output ***y***. If the vibration is measured at VS, perform equal-angle resampling to ***y*** according to the input RF signal *f_r_*(*t*). Perform the Hilbert transform (HT) followed by fast Fourier transform (FFT) to obtain the (order) envelope spectrum of the output signal ***y***.
6. Determine the health status or fault types of the bearing by investigating the (order) envelope spectrum.

### 3.4. Selection of Parameters

Filter length is one of the key parameters of FEAD. To guarantee that the FIR filter can cover the whole fault frequency band, the length of filter *L* is recommended [23]:

(33) $$L>2\frac{f_{s}}{f_{c}},$$

where *f_s_* is the sampling frequency of the signal, and *f_c_* is the resonance frequency of the bearing system. At the same time, the filter length should be as short as possible for better filtering performance, because a long filter length will increase the computational complexity and make it easier to deconvolute a single impulse [12]. In practice, the resonant frequency of the bearing system can reach to several thousand hertz, and the ratio of the sampling frequency to the resonant frequency is usually smaller than 10. Therefore, the filter size is selected as 20 in this work.

The weights *α* and *β* in FEAD control the importance of the FuzzyEn term and kurtosis term in the deconvolution process, respectively. A large α value makes FEAD stronger at suppressing noise interference in the output signal. However, it is prone to deconvoluting a single impulse and harmonic interference. A large *β* value makes FEAD filter out the fault impulses more easily while sacrificing the denoising performance. Since the FuzzyEn term in FEAD plays an auxiliary role in suppressing noise interference, *α* should take a smaller value relative to *β*. For convenience, this paper fixes *β* to 1 and adjusts *α* to achieve a balance between fault impulses enhancement and noise suppression. The proper value of *α* is recommended between 0.1 and 0.3 after trial and error.

In the SPSO-2011 algorithm, the value of each dimension in the particles is limited to [−1, 1] to ensure the symmetry of the filter coefficients. The number of iterations *T* is set to 100 to ensure that the result converge to the global optimal as much as possible.

## 4. Simulation Analysis

In this section, constant and variable speed simulation signals based on the vibration model in Section 3 are employed to verify the feasibility of the proposed FEAD method.

### 4.1. Case 1: Constant Speed Condition

**Figure 4:**
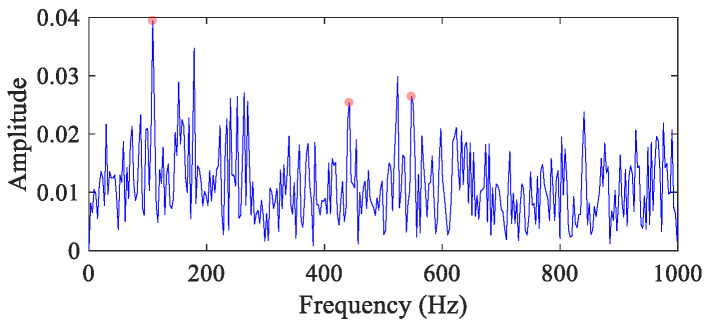
The envelope spectrum of the CS simulation signal (the red dots mark the fault feature peaks herein and after).

In this case, the bearing operates at constant RF 20 Hz, and the FCF is 110 Hz. The standard deviation of the Gaussian white noise is 0.35, with *SNR* = −11.27 dB. The time domain waveform has been analyzed in Figure 1g. Figure 4 displays the envelope spectrum of the original vibration signal. Affected by noise, only the peaks at 1×, 4× and 5× *FCF* (the fault feature peaks are marked with red dots herein and after) are pronounced.

**Figure 5:**
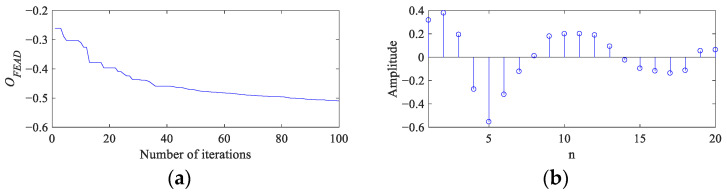

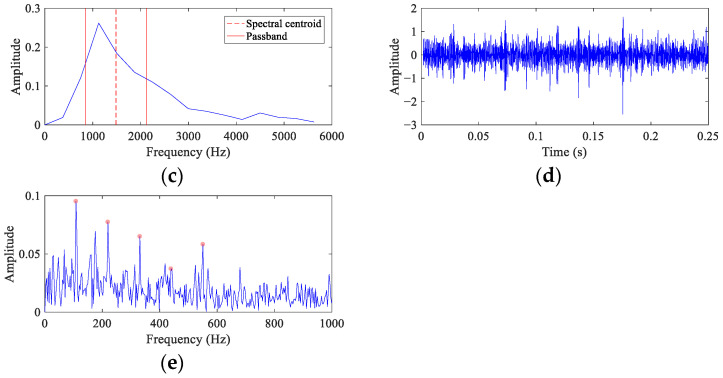
The process and results of FEAD for the CS simulation signal. (**a**) The fitness curve; (**b**,**c**) the optimal filter coefficients and its spectrum; and (**d**,**e**) the filtered signal and its envelope spectrum.

Because the SNR of CS simulation signal is very low, the value of *α* is set as 0.25 to make FEAD stronger in enhancing the fault features and suppressing the noise interference. Figure 5 presents the process and results of the FEAD method. The fitness curve (Figure 5a) indicates that the OF value changes smoothly after 50 iterations. The optimal filter coefficients and its spectrum are shown in Figure 5b and Figure 5c, respectively. The spectral centroid (indicated by a red dotted line) of the filter is 1486 Hz, which is close to the resonance frequency of the simulated signal. The frequency standard deviation is 641 Hz, and the corresponding passband (indicated by two red solid lines) of the filter is about 800 to 2100 Hz, which can precisely allow the resonant frequency band to pass through and suppress the components and noise in other frequency bands. Figure 5d depicts the signal filtered by FEAD, and noticeable fault impulses can be observed in the time domain. Its envelope spectrum with clear peaks at the first 5 FCF is shown in Figure 5e, which can serve as strong evidence of a bearing fault. Compared with Figure 4, FEAD enhances the fault features and suppresses noise in the signal envelope spectrum significantly.

### 4.2. Case 2: Variable Speed Condition

**Figure 6:**
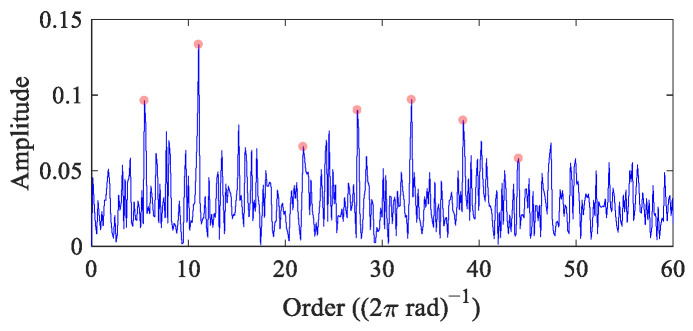
The order envelope spectrum of the VS simulation signal.

In this case, the RF of the bearing increases linearly from 18 Hz to 22 Hz. The FCC of the bearing is 5.5. To verify the performance of FEAD under strong noise interference, Gaussian white noise with a standard deviation of 0.9 is added to the simulated signal, with *SNR* = −10.32 dB. The time domain waveform is shown in Figure 1h. Figure 6 displays its order envelope spectrum, with fault peaks at 1×, 2× and 4~8× *FCC*. Although a bearing fault can be judged from this order envelope spectrum, the amplitudes of fault peaks are not noticeable enough at a high noise level.

**Figure 7:**
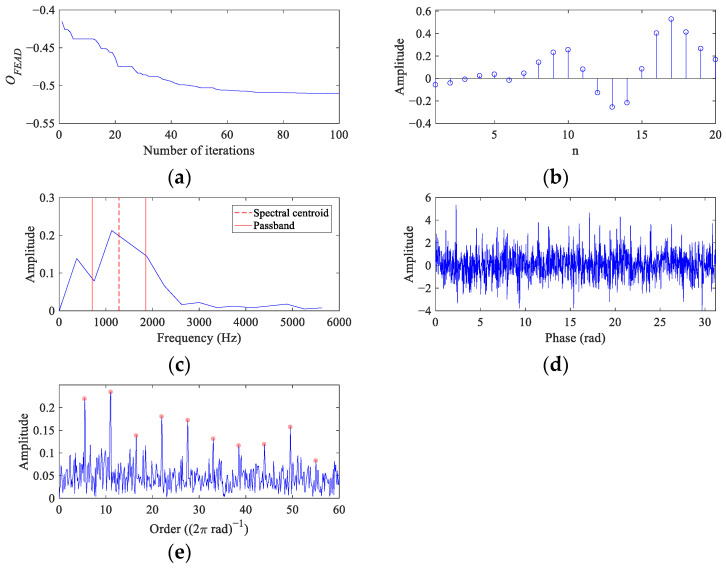
The process and results of FEAD for the VS simulation signal. (**a**) The fitness curve; (**b**,**c**) the optimal filter coefficients and its spectrum; and (**d**,**e**) the filtered signal in the angular domain and its order envelope spectrum.

Since the SNR of the VS simulation signal is higher than that of CS simulation signal, the value of *α* is set as 0.1 in FEAD to avoid deconvolving out a single impulse and harmonics. Figure 7 presents the process and results of FEAD for the VS simulation signal. The fitness curve in Figure 7a shows a stable trend after about 70 iterations, indicating that the filter solved by PSO is close to the global optimal. The optimal filter coefficients and its spectrum are displayed in Figure 7b and 7c, respectively. The spectral centroid and the frequency standard deviation reveal that the passband of the filter is approximately [700, 1850] Hz, which covers the resonance frequency band. However, signal components below 700 Hz can also partially pass through, indicating that the FuzzyEn term in the FEAD’s OF carries the risk of deconvoluting harmonic components, which verifies the analysis in Section 3.2. Nevertheless, the filtered signal in the angular domain (Figure 7d) presents clear fault impulses, and fault features from 1~10× *FCC* are dominant in its order envelope spectrum (Figure 7e). From this, a more firm conclusion that a fault exists in the bearing simulation signal can be drawn.

### 4.3. Comparison and Quantitative Evaluation

To further demonstrate the improvement of FEAD over the popular BD methods, MEDA, PSO-MEDA, MCKD and MOMEDA are employed to analyze the same simulation signals for comparison. To make a fair comparison, their filter lengths are selected as 20. The input period for MCKD and MOMEDA are set as *fs*·*T* and *fsp*/*FCC* (*fsp* is the angular resampling rate) for the CS and VS signals, respectively. The shift order of MCKD is selected as three to obtain as many impulses as possible. A rectangular window with a length of five is used in MOMEDA to reduce the error caused by bearing slippage.

**Figure 8:**
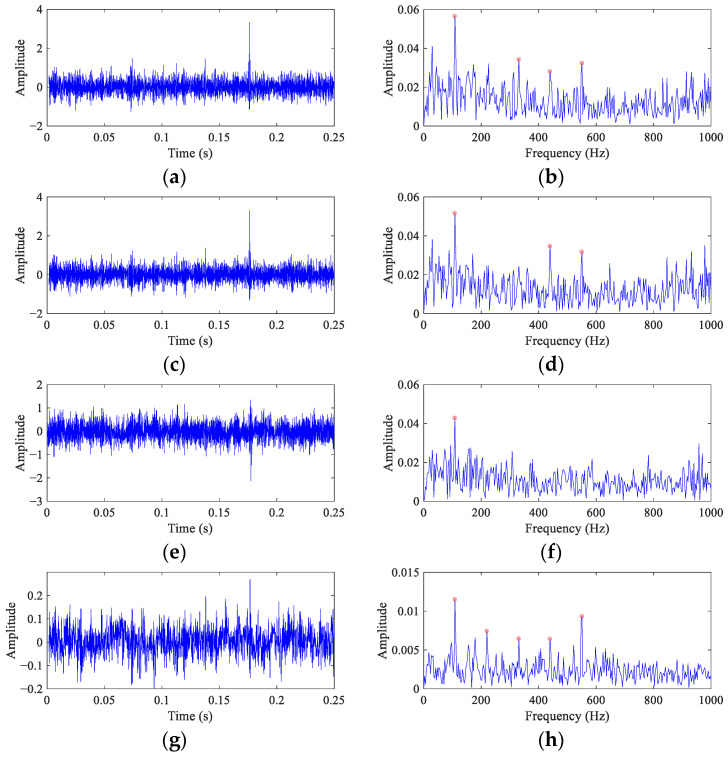
The filtered signals and their envelope spectra from the CS simulation signal using comparison methods. (**a**,**b**) MEDA; (**c**,**d**) PSO-MEDA; (**e**,**f**) MCKD; and (**g**,**h**) MOMEDA.

The filtered signals and their envelope spectra from the CS simulation signal using comparison methods are represented in Figure 8. The filtered signals from MEDA (Figure 8a) and PSO-MEDA (Figure 8c) show similar waveforms with random impulses, which exposes the shortcomings of the kurtosis-based BD methods. The envelope spectra of the MEDA (Figure 8b) and the PSO-MEDA (Figure 8d) filtered signals display comparable fault features. However, they are not as remarkable as those in the envelope spectrum of the FEAD filtered signal (Figure 5e). The reason that the fault feature peaks in the PSO-MEDA result is less than those in the MEDA result may be that the filter is not converging to the global optimal in the PSO iteration. This indicates that PSO is not the main reason for the improvement of FEAD compared to MEDA, but the new OF proposed in FEAD. Fault impulses cannot be observed in the filtered signal from MCKD (Figure 8e); only one peak appears at 1× *FCC* in its envelope spectrum (Figure 8f). On the contrary, there are clear periodic impulses in the filtered signal from MOMEDA (Figure 8g), as fault feature peaks at 1~5× *FCF* can also be distinguished in its envelope spectrum (Figure 8h).

In order to quantitatively evaluate the performance of FEAD and comparison methods, a fault feature index is proposed based on the envelope spectrum. The fault feature index for CS can be calculated as follows:

(34) $$I_{c}(N,c,M)=\frac{1}{N}\sum_{j=1}^{M}\frac{A(f_{m,j})}{mean(A(f_{m,j}-cFCF),A(f_{m,j}+cFCF))},$$

where *N* represents the number of different fault feature peaks observed in the envelope spectra obtained using all methods. *f_m_*_,*j*_ is the *j*th (*j* = 1, 2, …, *M*, *M* ≤ *N*) fault feature frequency where a distinguishable peak can be observed, and *A*(*f_m_*_,*j*_) is the amplitude at frequency *f_m_*_,*j*_. The denominator in the summation sign represents the mean amplitude of all spectral lines within the interval ***D*** = [*f_m_*_,*j*_ − *cFCC*, *f_m_*_,*j*_ + *cFCC*], where *c* is the proportional coefficient that control the interval width. The value of *c* needs to ensure that there is only one fault feature peak at *f_m_*_,*j*_, while no other fault feature peaks can be found in the interval ***D***. The ratio can reflect the identifiability of the fault peak at *f_m_*_,*j*_.

Similarity, the fault feature index for VS condition can be calculated as follows:

(35) $$I_{v}(N,c,M)=\frac{1}{N}\sum_{j=1}^{M}\frac{A(O_{m,j})}{mean(A(O_{m,j}-cFCC),A(O_{m,j}+cFCC))}$$

where *O_m_*_,*j*_ is the *j*th fault feature order where a distinguishable peak can be observed. The larger the value of the fault feature index, the more prominent or the greater the number of fault peaks in the (order) envelope spectrum. Therefore, the fault feature index can evaluate the diagnostic performance of different BD methods.

For the CS simulation signal, there are five different fault peaks in all the envelope spectra. So, let *N* = 5 and *c* = 0.1, and the *I_c_* values for different methods are calculated in Table 4.

It can be concluded from Table 4 that the *I_c_* value of the direct envelope is 1.01. The *I_c_* values of MEDA and PSO-MEDA are very close and higher than direct envelope, meaning that their diagnostic performance is improved. The *I_c_* value of MCKD is only 0.41, which is lower than the direct envelope. This indicates that MCKD does not enhance the fault impulses. Instead, the slippage error is enlarged due to the order shift in the correlated kurtosis calculation. The *I_c_* value of MOMEDA reaches 1.93, which is only lower than that of FEAD. This benefits from the application of the window function, which alleviates the interference of slippage error. The fault feature index of the proposed FEAD reaches the highest value of 2.16 among all methods, confirming that FEAD has the best performance in fault feature enhancement.

**Figure 9:**
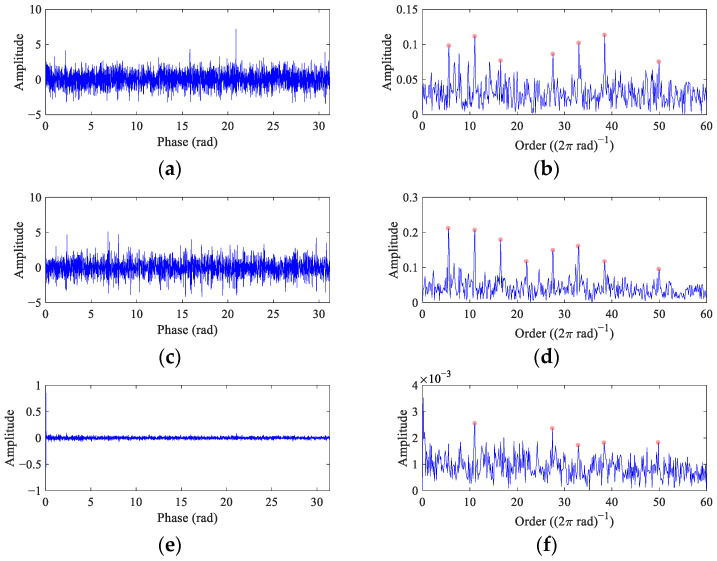

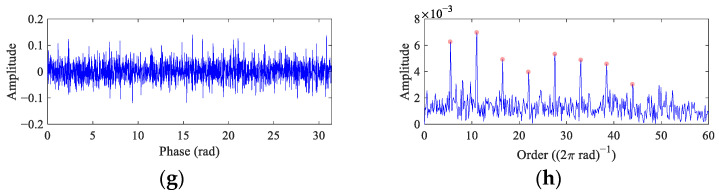
The filtered signals in the angular domain and their envelope spectra from the VS simulation signal using comparison methods. (**a**,**b**) MEDA; (**c**,**d**) PSO-MEDA; (**e**,**f**) MCKD; and (**g**,**h**) MOMEDA.

Figure 9 displays the comparison results for the VS simulation signal. There is a large random impulse in the angular domain of the MEDA filtered signal (Figure 9a), and its envelope spectrum (Figure 9b) shows similar characteristics to Figure 6, both of which present seven fault peaks. The fault impulses can be found in the PSO-MEDA filtered signal (Figure 9c), and its envelope spectrum (Figure 9d) shows eight fault peaks with a low noise level, indicating a significant improvement compared to MEDA. Fault features can hardly be found in the angular domain and the order envelope spectrum from MCKD (Figure 9e,f). Figure 9g illustrates the MOMEDA filtered signal, and it is not as sparse as that obtained by FEAD (Figure 7d). The corresponding order envelope spectrum displays distinct peaks at 1~8× FCC, which exhibits a diagnostic performance close to FEAD (Figure 7e).

For the VS simulation signal, there are 10 different fault peaks in all the order envelope spectra. So, let *N* = 10, *c* = 0.1, and the *I_v_* values for different methods are calculated in Table 5.

Conclusions similar to those in Figure 9 can be drawn from Table 5. The *I_v_* value of MEDA is slightly lower than direct order envelope analysis, suggesting that the fault features are not enhanced by MEDA. The *I_v_* value of PSO-MEDA is slightly improved compared to MEDA, which is consistent with the result shown in their order envelope spectra (Figure 9b,d). MCKD obtains the lowest *I_v_* value and the worst diagnostic performance because the resampling process further increases the period error. MOMEDA obtains the second largest *I_v_* value, 2.02. As expected, the FEAD method obtains the highest fault feature index value, which confirms that FEAD has the best diagnostic performance among all methods.

## 5. Experimental Validation

In this section, bearing test signals from the CWRU bearing date center, the railway wheelset and the EMU transmission test bench are used to validate the reliability and the effectiveness of the proposed FEAD method in practical applications.

### 5.1. CWRU Bearing Ball Fault

The CWRU bearing test bench consists of a motor, a torque transducer, a dynamometer, and control electronics. The bearing vibration data and a more detailed experimental introduction can be obtained from [37]. Ball faults of a bearing are considered the most difficult to diagnose [38]. Therefore, the bearing vibration signal with a ball fault is used to verify the reliability of the proposed FEAD. The motor drive end bearing ball fault data, with the name “X050_DE_time”, at a 12 kHz sampling frequency is selected as the example for analysis. The bearing model is 6205-2RS, and the depth of the fault is 0.028 inches. The vibration signal was collected at a constant speed of 1750 rpm, the corresponding theoretical FCF of the ball fault is 68.7 Hz, and the cage rotation frequency is 11.6 Hz.

**Figure 10:**
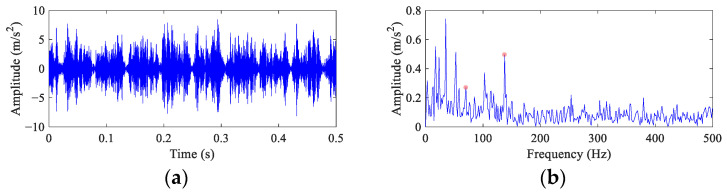
The vibration waveform and the envelope spectrum of the CWRU bearing ball fault data. (**a**) Time domain waveform and (**b**) envelope spectrum.

The time domain waveform and envelope spectrum of the vibration signal from the first 6000 samples are shown in Figure 10. The time domain waveform (Figure 10a) exhibits impulse feature, but it is difficult to find the continuous impulses excited by the ball fault with 1/*FCF* time intervals. The envelope spectrum (Figure 10b) shows peaks at *FCF* and 2× *FCF*. However, affected by noise, the peaks at *FCF* are very weak. At the low frequency band of the envelope spectrum, there are dominant harmonic peaks of the cage rotational frequency.

Since there are some fault features in the original envelope spectrum, in order to avoid deconvoluting out the cage rotational harmonics, the α value of FEAD is set to 0.1 for noise reduction and fault feature enhancement. The other BD methods mentioned in the simulation part are also used to process the same ball fault signal for comparative analysis, and the parameter settings of comparative BD methods are same as those in Section 4.3 herein and thereafter.

**Figure 11:**
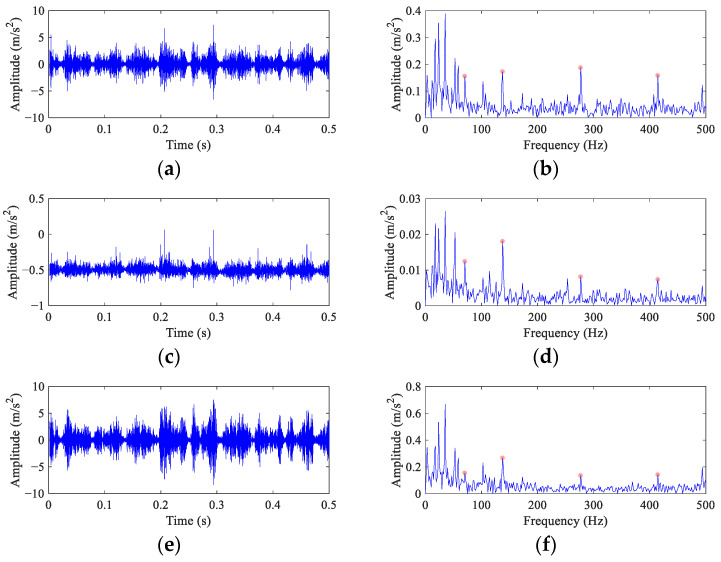

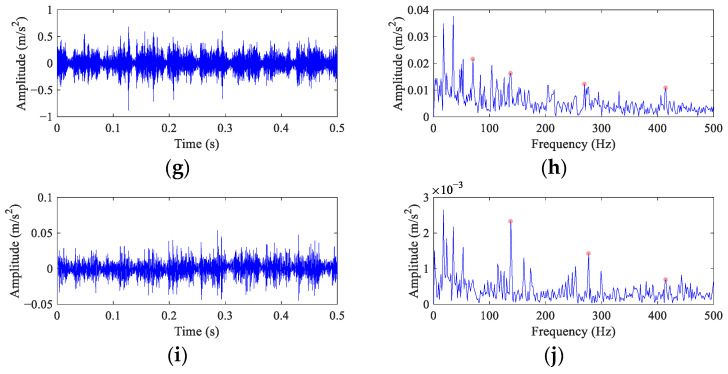
The filtered signals and their envelope spectra from the CWRU bearing ball fault signal using different BD methods. (**a**,**b**) FEAD; (**c**,**d**) MEDA; (**e**,**f**) PSO-MEDA; (**g**,**h**) MCKD; and (**i**,**j**) MOMEDA.

Figure 11 illustrates the results of FEAD and comparative BD methods. Compared with the original signal, the time domain waveforms of the deconvolved signals (the left column of Figure 11) obtained by these five BD methods are more sparse. It suggests that the impulses components are enhanced after deconvolution. However, it is still almost impossible to directly find the continuous equally spaced impulses caused by the ball fault from these time domain waveforms. Their envelope spectra are shown in the right column of Figure 11. In the envelope spectrum of the FEAD filtered signal (Figure 11b), a peak at *FCF* can be observed. In addition, peaks at even harmonics, i.e., 2×, 4× and 6× *FCF*, are also distinct, which is consistent with the ball fault characteristics described in [38]. Compared with Figure 10a, FEAD significantly enhances the ball fault features in the envelope spectrum. The envelope spectra of the MEDA and PSO-MEDA filtered signals (Figure 11d,f) both display four fault feature peaks, but the peaks at high FCF harmonics are not significant enough compared with those in FEAD. The envelope spectrum of the MCKD filtered signal (Figure 11h) also shows four fault characteristic peaks, but they are seriously interfered by strong noise. Figure 11j depicts the envelope spectrum of the MOMEDA filtered signal, and although the peak at 2× *FCF* is significant, no peak can be observed at *FCF* and the fault peak at 6× *FCF* is very weak.

In order to quantitatively analyze the above comparison results, the fault feature index of the envelope spectra are calculated based on Equation (34). There are four different FCFs in all envelope spectra, and so the *I_c_* values are calculated in Table 6 with *N* = 4 and *c* = 0.1.

The direct envelope method obtains the lowest *I_c_* value among all methods, which illustrates the necessity of enhancing the fault features of the original signal. The *I_c_* value of MEDA is two times higher than that of the direct envelope, which is the second highest among all methods, indicating that the feature enhancement effect of MEDA is significant. The *I_c_* value of PSO-MEDA is slightly lower than that of MEDA, which is consistent with the results shown by their envelope spectra. Although the *I_c_* values of MCKD and MOMEDA are higher than direct envelope, they are lower than other BD methods. The possible reason is that the slippage of rolling elements causes an inaccurate estimation of the failure period. The *I_c_* value of FEAD reaches 2.83, which is the highest among all methods. The result confirms that FEAD performs well in fault feature enhancement and can improve the reliability of the bearing ball fault diagnosis.

### 5.2. Wheelset Bearing Inner Race Fault at CS

**Figure 12:**
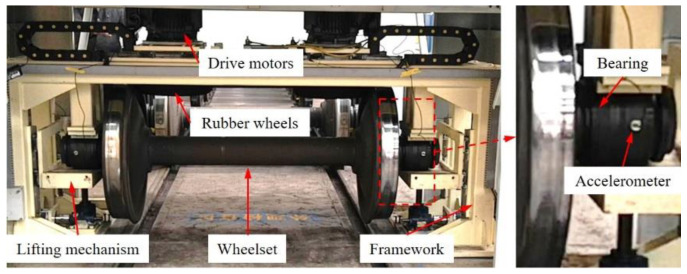
The wheelset bearing detection device.

Fault detection of wheelset bearings is an important step in the maintenance process of railway freight cars. At the maintenance site, vibration analysis is a key approach. The wheelset bearing vibration signal is collected through the detection device illustrated in Figure 12. The device mainly consists of a framework, lifting mechanism, drive motors and rubber wheels. The lifting mechanism lifts the wheelset and fixes the bearing outer race with the frame. The rubber wheels driven by motors rotate the wheelset to the rated speed of 293 rpm. Two YD-181 accelerometers are magnetically attached to the bearing outer race to collect vibration signals with a sampling frequency of 8000 Hz. The wheelset bearing model is 3532226X2-2RZ, and its geometric parameters and inner race FCF are listed in Table 7.

**Figure 13:**
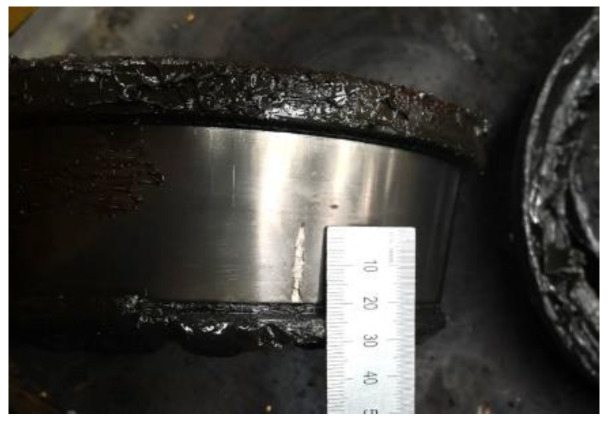
The wheelset bearing with spalling on the inner race.

During the detect process, the 1 s length bearing inner race fault vibration signal was collected. The faulty bearing is shown in Figure 13. A local spalling with a 20 mm length and 2 mm width can be found on the inner race.

**Figure 14:**
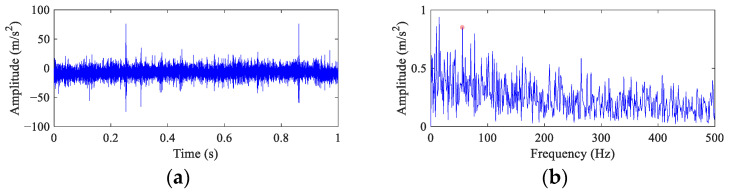
The vibration waveform and the envelope spectrum of the faulty wheelset bearing. (**a**) Time domain waveform and (**b**) envelope spectrum.

Figure 14 depicts the time domain waveform and the envelope spectrum of the original vibration signal. Since the spalling size is small, the fault impulses are submerged by noise. Only several random impulses with high amplitudes can be observed due to the external interference. There is only one blurry peak at 1× *FCF* in the envelope spectrum, which can hardly provide direct evidence of an inner race fault. In addition, the peaks at *f_r_* and its harmonics are dominant at the low frequency band.

**Figure 15:**
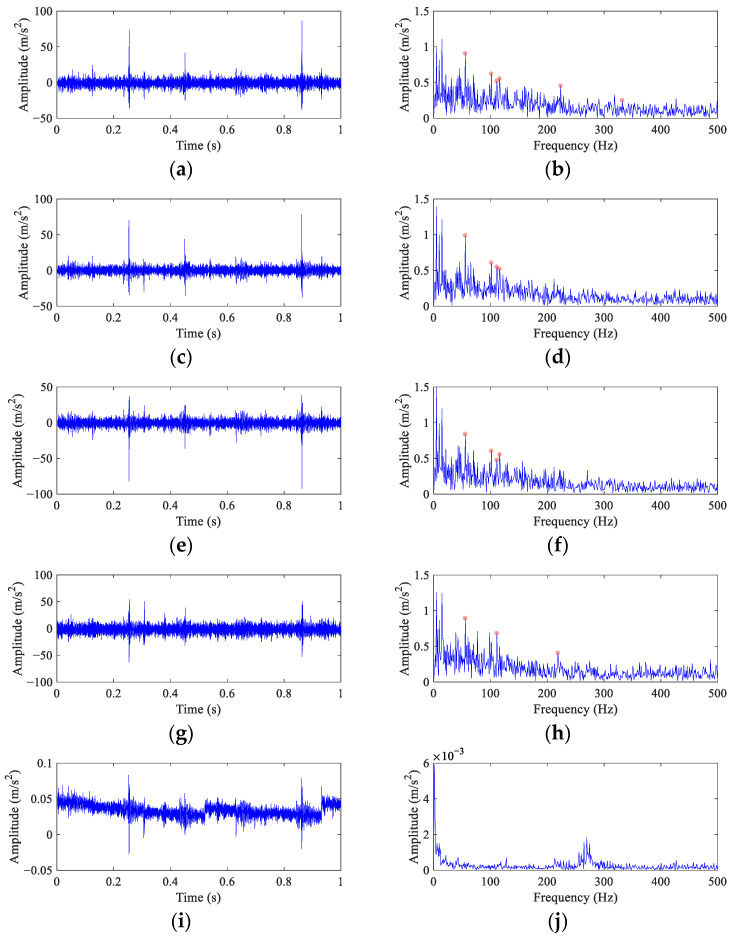
The filtered signals and their envelope spectra from the faulty wheelset bearing signal using different BD methods. (**a**,**b**) FEAD; (**c**,**d**) MEDA; (**e**,**f**) PSO-MEDA; (**g**,**h**) MCKD; and (**i**,**j**) MOMEDA.

Because the fault peak can be found in the original envelope spectrum even though it is weak, the *α* in FEAD is selected as 0.1 to avoid filtering out the rotational harmonics. At the same time, four other BD methods are employed for comparison. The results of FEAD and comparison methods are presented in Figure 15. The random impulses are retained in the filtered signals using these five BD methods. It is nearly impossible to identify faults through their time domain waveforms (the left column). The envelope spectrum of the FEAD filtered signal (Figure 15b) displays distinct peaks at 1×, 4× and 6× *FCF*. Meanwhile, peaks at 2× *FCF* and its modulated frequencies, i.e., 2*FCF* − 2*f_r_* and 2*FCF* + *f_r_* can also be observed, which fully demonstrates that fault is caused by inner race damage. In comparison, the envelope spectrum of the MEDA (Figure 15d) and the PSO-MEDA (Figure 15f) filtered signals do not show peaks at the higher harmonics of FCF. Fault peaks in the envelope spectrum obtained by the MCKD (Figure 15h) appear at 1×, 2× and 4× *FCF*. The fault information in MCKD is less than that in FEAD (Figure 15b). The waveform of the filtered signal using MOMEDA (Figure 15i) shows an unstable trend. The corresponding envelope spectrum (Figure 15j) does not show any fault characteristics.

The fault feature index *I_c_* is employed to quantitatively evaluate the performance of different methods. The number of the different FCFs in all the envelope spectra is six. To distinguish the 2× *FCF* and its modulated frequencies, the proportion coefficient *c* is selected as 0.05. The *I_c_* values of different methods are calculated in Table 8.

The conclusions drawn from Table 8 are consistent with those from Figure 15. The *I_c_* value of direct envelope is only 0.30. MEDA and PSO-MEDA have the similar capabilities in fault feature enhancement, because their *I_c_* values are close. The *I_c_* values of MCKD and MOMEDA suggest that they have completely opposite performances. This is contrary to the results from the simulation part, and it may be that the degree of the wheelset bearing slippage is smaller than that in the simulation signal. However, the window function in MOMEDA introduces an extra error when the fault period is stationary. The *I_c_* value of FEAD is the highest at 1.87 among all the methods, which validates the good performance in the wheelset bearing fault diagnosis.

### 5.3. Test Bench Bearing Outer Race Fault at VS

**Figure 16:**
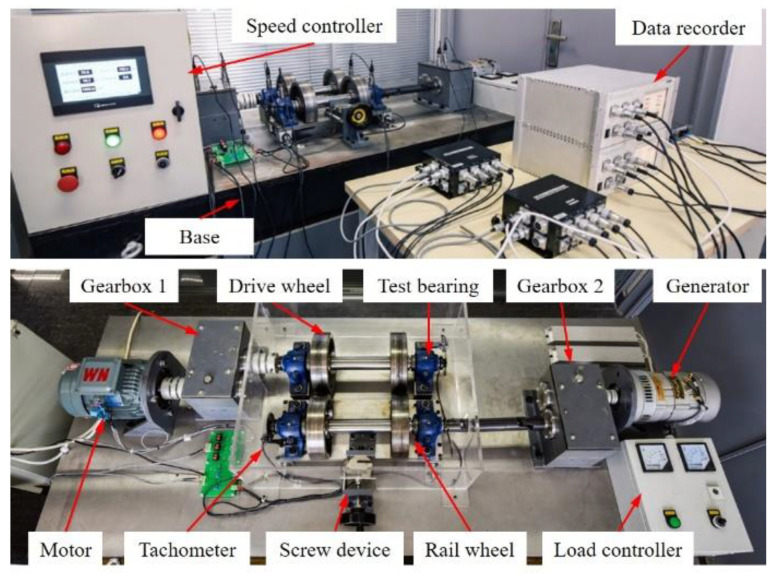
The EMU transmission test bench.

In this section, the performance of FEAD at VS is validated by the bearing outer race fault signal from the EMU transmission test bench. Figure 16 illustrates the components of test bench; it simulates the power transmission process of the EMU from the traction motor to the rails. The motor transmits the power to the drive wheels through a parallel helical gearbox 1. The drive wheels are supported by two double row tapered roller bearings with model 351306, and its geometric parameters and outer race FCO are listed in Table 9. The drive wheels contact with the rail wheels with their contact force adjusted by a screw device. The rail wheels are connected to the generator through the universal joint shaft and the gearbox 2 to simulate the resistance when the EMU is running.

**Figure 17:**
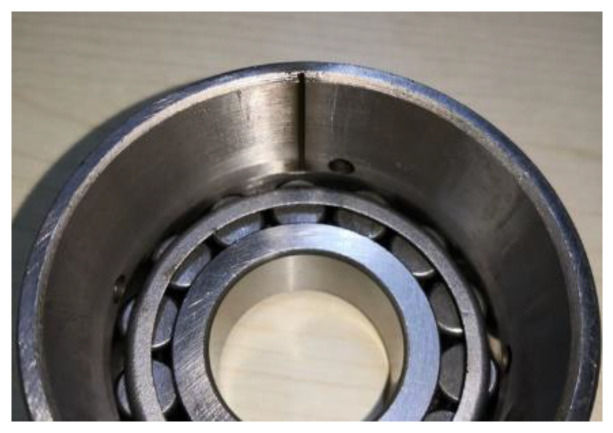
The test bench bearing with a fault on the outer race.

The bearing on the right drive wheel was seeded with an outer race fault using electro-discharge machining. The fault width is 1 mm, as shown in Figure 17. The accelerometer with model BHT01-100 is bolted onto the bearing seat. The 1 s length vibration and speed pulse of the test bearing are collected simultaneously with an 8000 Hz sampling frequency.

**Figure 18:**
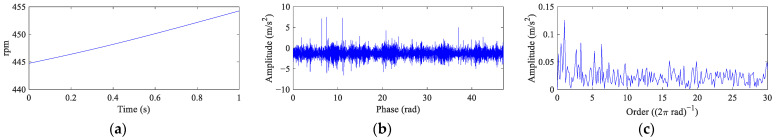
The speed curve (**a**), the vibration waveform in the angular domain (**b**), and the order envelope spectrum (**c**) of the test bench faulty bearing.

The speed curve, the vibration waveform in the angular domain and the order envelope spectrum are shown in Figure 18. The speed increases from 444.8 rpm to 454.3 rpm in an approximately linear manner. Although several impulses occur in the angular domain waveform (Figure 18b), no fault-related peaks can be observed in the order envelope spectrum (Figure 18c). Conversely, the order envelope spectrum is dominated by the rotational order.

**Figure 19:**
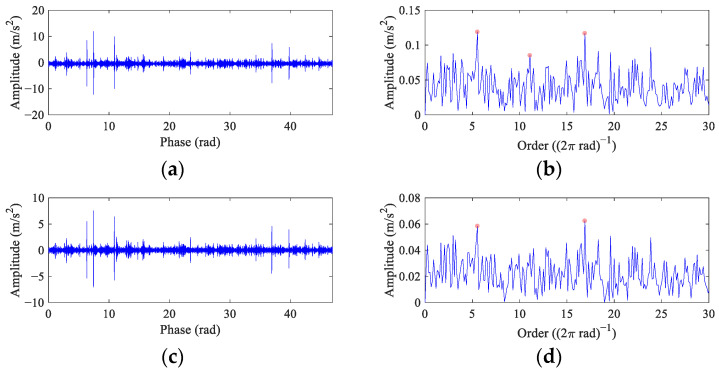

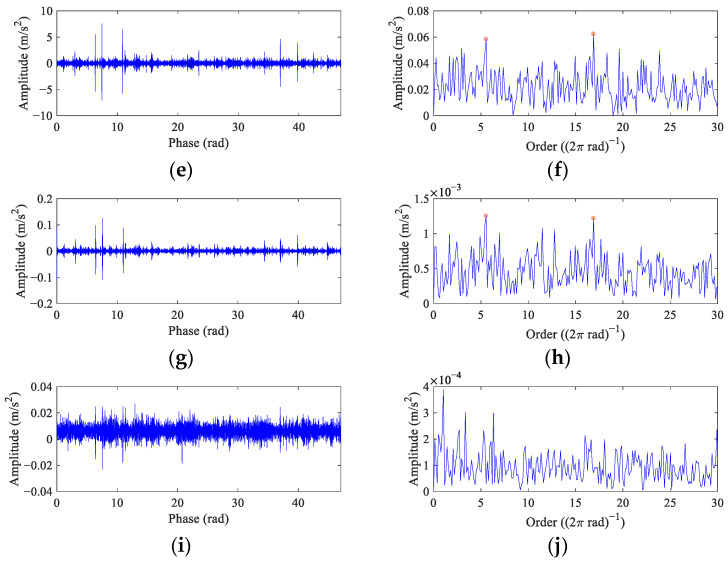
The filtered signals in the angular domain and their envelope spectra from the test bench bearing signal using different BD methods. (**a**,**b**) FEAD; (**c**,**d**) MEDA; (**e**,**f**) PSO-MEDA; (**g**,**h**) MCKD; and (**i**,**j**) MOMEDA.

Since the original order envelope spectrum is dominated by rotational harmonics and no fault-related peaks can be found in it, *α* is selected as 0.2 in FEAD to strike a balance between enhancing fault features and preventing it from deconvolving harmonic signals. The results of FEAD and comparison methods are shown in Figure 19. Although random impulses still exist in the FEAD filtered signal (Figure 19a), it is sparser than the original signal and the fault impulses are enhanced. The fault peaks at 1~3× *FCO* in the order envelope spectrum (Figure 19b) suggest that a fault exits on the bearing outer race. Even though the waveforms of the MEDA, the PSO-MEDA and the MCKD output (Figure 19c,e,g) are similar to that of the FEAD, the peaks at 2× *FCO* are missed in their envelope spectra (Figure 19d,f,h). It is worth noting that the peak at the 11.48 order in Figure 19h cannot be treated as the fault feature because its relative error with 2× *FCO* exceeds 3% [39]. The outer race fault features cannot be identified in the MOMEDA output (Figure 19i,j). The reason may also lie in the window function.

There are three peaks at different FCOs in all the envelope spectra. Let *N* = 3 and *c* = 0.1; the *I_v_* values for different methods are calculated in Table 10. Since no fault feature can be found in the results from the direct envelope and MOMEDA, their *I_v_* values are zero. The fault feature index values of MEDA, PSO-MEDA and MCKD are close to each other, implying that these three BD methods have similar performance in fault enhancement. Once again, FEAD achieves the highest *I_v_* value among all methods, confirming its advantage on the fault feature enhancement under VS.

## 6. Conclusions

In this work, a novel blind deconvolution method, FEAD, is proposed for enhancing the weak fault impulses and eliminating the noise interferences in bearing vibration signals. Taking the advantage of FuzzyEn’s high sensitivity to noise and the PSO algorithm, the proposed FEAD method iteratively solves the optimal inverse FIR filter that minimizes the weighted FuzzyEn–kurtosis of the filtered signal. The feasibility of the proposed method is first verified based on the simulation signals. The simulation results confirm that FEAD has good performance in fault feature enhancement under constant and variable speed conditions, even in the low SNR condition. The FEAD method is further validated on the experimental signals. The ball fault diagnostic result from CWRU bearing data confirm the reliability of the proposed method. The diagnostic results from the railway wheelset bearing and the EMU test bench bearing signals suggest that FEAD can effectively detect the incipient spalling on the bearing inner race at CS and the outer race fault at VS. Comparative and quantitative studies with direct envelope, MEDA, PSO-MEDA, MCKD and MOMEDA draw the following conclusions:

1. The main reason for the improvement in FEAD lies in the noise suppression effect from the new objective function rather than the PSO algorithm.
2. MCKD and MOMEDA are susceptible to the estimated errors in fault period. Bearing slippage and equal-angle resampling will amplify these errors, while FEAD is immune to these impacts.
3. FEAD outperforms all the comparison methods in fault feature enhancement and noise elimination.

However, the proposed FEAD method still has some weaknesses and limitations. The low computational efficiency of fuzzy entropy makes the FEAD method time-consuming. Necessary attempts to control the number of calculations of fuzzy entropy in iterations should be taken to reduce the computational burden. The FIR filter of FEAD is still solved iteratively, and more efficient intelligent optimization methods can be tried to improve the convergence. More advanced entropy methods also need to be studied and incorporated into BD methods in future research.

## Figures and Tables

**Table 1:** Parameter values of the bearing vibration model.

**Component**	***b_c_*(*t*)**															
**Parameter**	*A_ic_*			*T*(s)			*T_t_*(s)				*β*_1_			*ω_r_*_1_ (Hz)		
**Values**	*U*(0.8, 1)			1/110			0.25				2000			1700		
**Component**	***b_v_*(*t*)**															
**Parameter**	*A_0_*	*η*			*f_r_*(*t*) (Hz)			*FCC*		*T_t_* (s)			*β*_1_			*ω_r_*_1_ (Hz)
**Values**	0.5	0.1			16t + 18			5.5		0.25			2000			1700
**Component**	***d*(*t*)**															
**Parameter**	*M*			*D_m_*			*β*_2_				*ω_r_*_2_ (Hz)			*T_m_* (s)		
**Values**	1			3			3000				2000			0.175		
**Component**	***h_c_*(*t*)**															
**Parameter**	*P*_1_		*h*_1_(*t*) (Hz)			*θ*_1_			*P*_2_			*h*_2_(*t*) (Hz)			*θ*_2_	
**Values**	0.05		20			π/6			0.2			400			−π/3	
**Component**	***h_v_*(*t*)**															
**Parameter**	*P*_1_		*h*_1_(*t*) (Hz)			*θ*_1_			*P*_2_			*h*_2_(*t*) (Hz)			*θ*_2_	
**Values**	0.05		*f_r_*(*t*)			π/6			0.2			20*f_r_*(*t*)			−π/3	
**Component**	***n*(*t*)**															
**Parameter**	Std for the CS model								Std for the VS model							
**Values**	0.35								0.9							

**Table 2:** Kurtosis and entropy values of different components and mixed signals at CS.

Components	*b_c_*(*t*)	*d*(*t*)	*h_c_*(*t*)	*n*(*t*)	*x_c_*(*t*)
Kurt	25.0	1009.0	1.7	3.0	3.2
FuzzyEn	0.0955	0.0035	0.0457	0.9132	0.8916
ApEn	0.1127	0.0030	0.2896	2.0141	2.0036
SampEn	0.0274	0.0010	0.3197	2.1881	2.1741

**Table 3:** Kurtosis and entropy values of different components and mixed signals under VS.

Components	*b_v_*(*t*)	*d*(*t*)	*h_v_*(*t*)	*n*(*t*)	*x_v_*(*t*)
Kurt	24.7	1009.0	1.7	3.0	3.4
FuzzyEn	0.1084	0.0035	0.0457	1.3321	1.3340
ApEn	0.1099	0.0030	0.2971	2.0206	1.9914
SampEn	0.0281	0.0010	0.3276	2.2001	2.1609

**Table 4:** The fault feature index values of different methods for the CS simulation signal.

Methods	Direct Envelope	MEDA	PSO-MEDA	MCKD	MOMEDA	FEAD
*I_c_*	1.01	1.40	1.38	0.41	1.93	2.16

**Table 5:** The fault feature index values of different methods for the VS simulation signal.

Methods	Direct Envelope	MEDA	PSO-MEDA	MCKD	MOMEDA	FEAD
*I_v_*	1.66	1.51	1.82	0.95	2.02	2.50

**Table 6:** The fault feature index values of different methods for the CWRU bearing signal.

Methods	Direct Envelope	MEDA	PSO-MEDA	MCKD	MOMEDA	FEAD
*I_c_*	1.28	2.66	2.31	1.98	2.11	2.83

**Table 7:** The geometric parameters and inner race FCF of the wheelset bearing.

Roller Diameter (mm)	Pitch Diameter (mm)	Contact Angle (degree)	Number of Rollers (Signal Row)	Inner Race FCF (Hz)
23.7	179.5	8.83	20	55.3

**Table 8:** The fault feature index values of different methods for the wheelset bearing signal.

Methods	Direct Envelope	MEDA	PSO-MEDA	MCKD	MOMEDA	FEAD
*I_c_*	0.30	1.30	1.23	1.00	0	1.87

**Table 9:** The geometric parameters and outer race FCO of the test bench bearing.

Roller Diameter (mm)	Pitch Diameter (mm)	Contact Angle (Degree)	Number of Rollers (Signal Row)	Outer Race FCO
10.59	51.21	25.5	14	5.57

**Table 10:** The fault feature index values of different methods for the test bench bearing signal.

Methods	Direct Envelope	MEDA	PSO-MEDA	MCKD	MOMEDA	FEAD
*I_v_*	0	1.29	1.26	1.21	0	1.89

## Data Availability

The experimental bearing data are available on request from the corresponding author.

## Nomenclature

*A_i_*	amplitude of the *i*th impulse in the signal model	OF	objective function in the BD method
*A*_0_	a constant amplitude in *A_i_*	*P*	amplitude of the harmonic in the signal model
BD	blind deconvolution	*T*	time interval between two adjacent fault impulses
*b_c_*(*t*)	fault impulses in the constant speed signal model	*T_m_*	time of the *m*th random impulse
*b_v_*(*t*)	fault impulses in the variable speed signal model	*T_i_*	time of the *i*th fault impulse
CS	constant speed	*T* *_t_*	total duration of the simulation signal
*D*_*m*_	amplitude of the *m*th random impulse	*u*(‧)	unit step function in the signal model
*d*(*t*)	random impulses in the signal model	VS	variable speed
FCC	fault characteristic coefficient	*x*(*t*)	mixed signal in the bearing vibration model
FCF	fault characteristic frequency	*ω*_*r*1_	resonant frequency excited by fault impulses
*f_r_*(*t*)	function of rotational frequency	*ω*_*r*2_	resonant frequency excited by random impulses
FuzzyEn	fuzzy entropy	*β*_1_	decay parameter of fault impulses
*h*(*t*)	discrete harmonics in the signal model	*β*_2_	decay parameter of random impulses
*I_c_*	fault feature index for the constant speed signal	*θ*	initial phase of the harmonic in the signal model
*I_v_*	fault feature index for the variable speed signal	*τ*	time error of the impulses caused by bearing slippage
*M*	number of random impulses	*η*	proportional coefficient of amplitude in *A_i_*
*n*(*t*)	noise in the signal model		

## References

[1] Miao Y., Zhang B., Lin J., Zhao M., Liu H., Liu Z., Li H. (2022). A review on the application of blind deconvolution in machinery fault diagnosis. Mech. Syst. Signal Process..

[2] Chen J., Li Z., Pan J., Chen G., Zi Y., Yuan J., Chen B., He Z. (2016). Wavelet transform based on inner product in fault diagnosis of rotating machinery: A review. Mech. Syst. Signal Process..

[3] Lei Y., Lin J., He Z., Zuo M.J. (2013). A review on empirical mode decomposition in fault diagnosis of rotating machinery. Mech. Syst. Signal Process..

[4] Dragomiretskiy K., Zosso D. (2014). Variational Mode Decomposition. IEEE Trans. Signal Process..

[5] Chen S., Dong X., Peng Z., Zhang W., Meng G. (2017). Nonlinear Chirp Mode Decomposition: A Variational Method. IEEE Trans. Signal Process..

[6] Antoni J. (2006). The spectral kurtosis: A useful tool for characterising non-stationary signals. Mech. Syst. Signal Process..

[7] Antoni J. (2007). Fast computation of the kurtogram for the detection of transient faults. Mech. Syst. Signal Process..

[8] Zhou K., Tang J. (2021). Harnessing fuzzy neural network for gear fault diagnosis with limited data labels. Int. J. Adv. Manuf. Technol..

[9] Lou X., Loparo K.A. (2004). Bearing fault diagnosis based on wavelet transform and fuzzy inference. Mech. Syst. Signal Process..

[10] Endo H., Randall R.B. (2007). Enhancement of autoregressive model based gear tooth fault detection technique by the use of minimum entropy deconvolution filter. Mech. Syst. Signal Process..

[11] McDonald G.L., Zhao Q., Zuo M.J. (2012). Maximum correlated Kurtosis deconvolution and application on gear tooth chip fault detection. Mech. Syst. Signal Process..

[12] McDonald G.L., Zhao Q. (2017). Multipoint Optimal Minimum Entropy Deconvolution and Convolution Fix: Application to vibration fault detection. Mech. Syst. Signal Process..

[13] Wang C., Li H., Ou J., Hu R., Hu S., Liu A. (2020). Identification of planetary gearbox weak compound fault based on parallel dual-parameter optimized resonance sparse decomposition and improved MOMEDA. Measurement.

[14] Cheng Y., Wang Z., Zhang W., Huang G. (2019). Particle swarm optimization algorithm to solve the deconvolution problem for rolling element bearing fault diagnosis. Isa Trans..

[15] Miao Y., Zhao M., Lin J., Xu X. (2016). Sparse maximum harmonics-to-noise-ratio deconvolution for weak fault signature detection in bearings. Meas. Sci. Technol..

[16] Buzzoni M., Antoni J., D’Elia G. (2018). Blind deconvolution based on cyclostationarity maximization and its application to fault identification. J. Sound Vib..

[17] Liang K., Zhao M., Lin J., Jiao J., Ding C. (2021). Maximum average kurtosis deconvolution and its application for the impulsive fault feature enhancement of rotating machinery. Mech. Syst. Signal Process..

[18] Hashim S., Shakya P. (2023). A spectral kurtosis based blind deconvolution approach for spur gear fault diagnosis. Isa Trans..

[19] Cheng Y., Zhou N., Zhang W., Wang Z. (2018). Application of an improved minimum entropy deconvolution method for railway rolling element bearing fault diagnosis. J. Sound Vib..

[20] Jia X., Zhao M., Di Y., Jin C., Lee J. (2017). Investigation on the kurtosis filter and the derivation of convolutional sparse filter for impulsive signature enhancement. J. Sound Vib..

[21] Wang Z., Du W., Wang J., Zhou J., Han X., Zhang Z., Huang L. (2019). Research and application of improved adaptive MOMEDA fault diagnosis method. Measurement.

[22] Cheng Y., Chen B., Zhang W. (2019). Adaptive Multipoint Optimal Minimum Entropy Deconvolution Adjusted and Application to Fault Diagnosis of Rolling Element Bearings. IEEE Sens. J..

[23] Miao Y., Zhao M., Lin J., Lei Y. (2017). Application of an improved maximum correlated kurtosis deconvolution method for fault diagnosis of rolling element bearings. Mech. Syst. Signal Process..

[24] Xu W., Tan H., Zhao M. (2022). An improved multipoint optimal minimum entropy deconvolution adjusted method for the diagnosis of rotating machinery under variable speed conditions. Proc. Inst. Mech. Eng. Part C J. Mech. Eng. Sci..

[25] Li Z., Ma J., Wang X., Wu J. (2019). MVMD-MOMEDA-TEO Model and Its Application in Feature Extraction for Rolling Bearings. Entropy.

[26] Sun R., Yang J., Yao D., Wang J. (2022). A New Method of Wheelset Bearing Fault Diagnosis. Entropy.

[27] He D., Wang X., Li S., Lin J., Zhao M. (2016). Identification of multiple faults in rotating machinery based on minimum entropy deconvolution combined with spectral kurtosis. Mech. Syst. Signal Process.

[28] Li Y., Wang X., Liu Z., Liang X., Si S. (2018). The Entropy Algorithm and Its Variants in the Fault Diagnosis of Rotating Machinery: A Review. IEEE Access.

[29] Leite G.D.N.P., Araújo A.M., Rosas P.A.C., Stosic T., Stosic B. (2019). Entropy measures for early detection of bearing faults. Phys. A Stat. Mech. Its Appl..

[30] Jiao J., Yue J., Pei D. (2022). Feature Enhancement Method of Rolling Bearing Based on K-Adaptive VMD and RBF-Fuzzy Entropy. Entropy.

[31] Chen W., Wang Z., Xie H., Yu W. (2007). Characterization of Surface EMG Signal Based on Fuzzy Entropy. IEEE Trans. Neural Syst. Rehabil. Eng..

[32] Zambrano-Bigiarini M., Clerc M., Rojas R. Standard Particle Swarm Optimisation 2011 at CEC-2013: A baseline for future PSO improvements. Proceedings of the 2013 IEEE Congress on Evolutionary Computation.

[33] Clerc M. Standard Particle Swarm Optimisation. http://clerc.maurice.free.fr/pso/SPSO_descriptions.pdf.

[34] Clerc M. Back to Random Topology. http://clerc.maurice.free.fr/pso/random_topology.pdf.

[35] Pei D., Yue J., Jiao J. (2023). A Novel Method for Bearing Fault Diagnosis under Variable Speed Based on Envelope Spectrum Fault Characteristic Frequency Band Identification. Sensors.

[36] Chen W., Zhuang J., Yu W., Wang Z. (2009). Measuring complexity using FuzzyEn, ApEn, and SampEn. Med. Eng. Phys..

[37] Case Western Reserve University Bearing Data Center Website. https://engineering.case.edu/bearingdatacenter.

[38] Smith W.A., Randall R.B. (2015). Rolling element bearing diagnostics using the Case Western Reserve University data: A benchmark study. Mech. Syst. Signal Process..

[39] Randall R.B., Antoni J. (2011). Rolling element bearing diagnostics—A tutorial. Mech. Syst. Signal Process..

